# Supplementary material for: Cytomegalovirus Specific Serological and Molecular Markers in a Series of Pregnant Women with Cytomegalovirus Non Primary Infection
Source: Viruses. 2022 Oct 31;14(11):2425. doi: 10.3390/v14112425 (PMC9698281; doi:10.3390/v14112425)
Supplement: Supplementary file 1 [file viruses-14-02425-s001.zip › viruses-1980444-supplementary.pdf]

Supplementary data

Figure S1: Number of serums according to term of pregnancy.

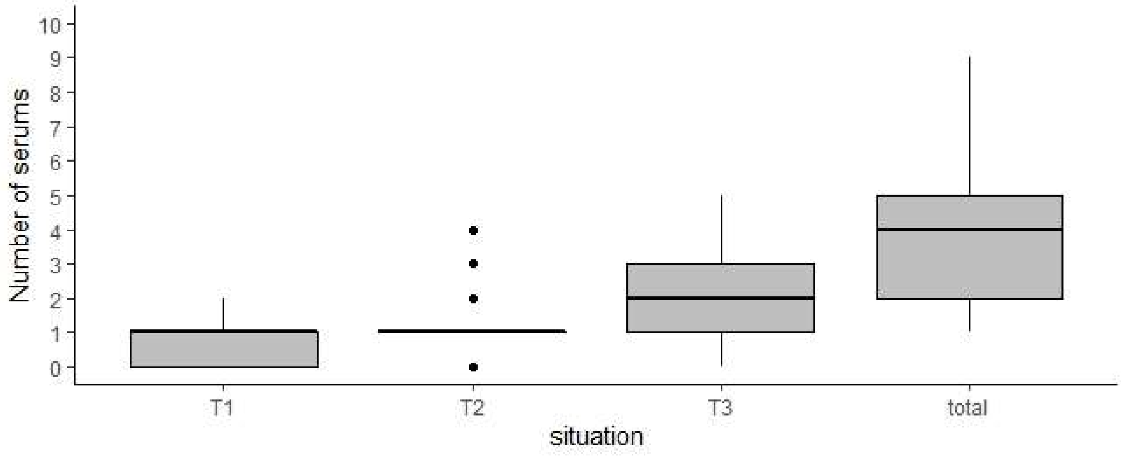

The boxplot shows the number of serum samples per patient, according to the term of pregnancy: first, second, third trimester and total.

T1: first trimester; T2: second trimester; T3: third trimester
